# Supplementary material for: “If we work as a team, there are success stories.” Unpacking team members’ perceptions and experiences of what impacts team performance in a maternal and neonatal quality improvement programme in South Africa, before, and during COVID-19
Source: PLOS Glob Public Health. 2024 Dec 23;4(12):e0003780. doi: 10.1371/journal.pgph.0003780 (PMC11665988; doi:10.1371/journal.pgph.0003780)
Supplement: S1 Table — (DOCX) [file pgph.0003780.s001.docx]

**S1 Table: Team member interview foci**

| - Training recall - How teams were established - Operationalising their QI work - Experiences of being a leader / member - Team performance, successes, and challenges - Enablers and barriers at facility and district level - COVID-19 impact on service delivery in general and team functioning |
| --- |
