# Supplementary material for: “If we work as a team, there are success stories.” Unpacking team members’ perceptions and experiences of what impacts team performance in a maternal and neonatal quality improvement programme in South Africa, before, and during COVID-19
Source: PLOS Glob Public Health. 2024 Dec 23;4(12):e0003780. doi: 10.1371/journal.pgph.0003780 (PMC11665988; doi:10.1371/journal.pgph.0003780)
Supplement: S2 Table — (DOCX) [file pgph.0003780.s002.docx]

**S2 Table. Consolidated criteria for reporting qualitative studies (COREQ): 32-item checklist**

**Manuscript title:** “If we work as a team, there are success stories.” Unpacking team members’ perceptions and experiences of what impact team performance in a maternal and neonatal quality improvement programme in South Africa, before, and during COVID-19

| **No. Item** | **Guide questions/description** | **Reported on Page #** |
| --- | --- | --- |
| **Domain 1: Research team and reﬂexivity** |  |  |
| *Personal Characteristics* |  |  |
| 1. Interviewer/facilitator | Which author/s conducted the interview or focus group? | WO  (p11, line 224) |
| 2. Credentials | What were the researcher’s credentials? E.g. PhD, MD | Holds a Master in  Research Psychology.  (p11, line 225) |
| 3. Occupation | What was their occupation at the time of the study? | Scientist at the  South African  Medical Research  Council.  (p11, line 224) |
| 4. Gender | Was the researcher male or female? | Male  (p11, line 224) |
| 5. Experience and training | What experience or training did the researcher have? | 18 years’ experience  of conducting  qualitative research.  (p11, line 225) |
| *Relationship with participants* |  |  |
| 6. Relationship established | Was a relationship established prior to study commencement? | No, but there was a  participant recruitment visit  conducted before the  data collection  commenced.  (p9, line 200) |
| 7. Participant knowledge of the interviewer | What did the participants know about the researcher? e.g. personal goals, reasons for doing the research | The information  detailed in the study  information letter.  (p9, lines 204-6) |
| 8. Interviewer characteristics | What characteristics were reported about the interviewer/facilitator? e.g. Bias, assumptions, reasons and interests in the research topic | None of his  characteristics were  regarded as bias  confounders. |
| **Domain 2: study design** |  |  |
| *Theoretical framework* |  |  |
| 9. Methodological orientation and Theory | What methodological orientation was stated to underpin the study? e.g. grounded theory, discourse analysis, ethnography, phenomenology, content analysis | We followed a thematic content analysis approach.  (p12, lines 254 - p13, line 263)  We also applied the Consolidated Framework for Implementation Research in analysing the data.  (p12, lines 258-60) |
| *Participant selection* |  |  |
| 10. Sampling | How were participants selected? e.g. purposive, convenience, consecutive, snowball | Purposive sampling of facilities.  (p8, lines 178-85)  The team leaders recruited the member members.  (p9, lines 200-04) |
| 11. Method of approach | How were participants approached? e.g. face-to-face, telephone, mail, email | Face-to-face  (p9, line 200) |
| 12. Sample size | How many participants were in the study? | 47  (p9, line 196) |
| 13. Non-participation | How many people refused to participate or dropped out? Reasons? | One facility declined participation (p8, lines 183-4); no members declined; there was no drop as most members were once interviewed (p10, lines 212-3) |
| *Setting* |  |  |
| 14. Setting of data collection | Where was the data collected? e.g. home, clinic, workplace | Health facility where the participants worked.  (p11, line 220-1) |
| 15. Presence of non-participants | Was anyone else present besides the participants and researchers? | No |
| 16. Description of sample | What are the important characteristics of the sample? e.g. demographic data, date | Most were professional nurses with a median of 23 and 18 years’ experience in nursing and working at the facility, respectively.  (p13, lines 274-9; Table 3) |
| *Data collection* |  |  |
| 17. Interview guide | Were questions, prompts, guides provided by the authors? Was it pilot tested? | Interview foci (Supporting information 1)  It was piloted within the research team.  (p11, lines 222-3) |
| 18. Repeat interviews | Were repeat interviews carried out? If yes, how many? | The data collection took place over three-time points.  (p9, lines 203-4) |
| 19. Audio/visual recording | Did the research use audio or visual recording to collect the data? | Audio recorded  (p11, line 224) |
| 20. Field notes | Were ﬁeld notes made during and/or after the interview or focus group? | The lead author kept a fieldwork journal.  (p11, line 230) |
| 21. Duration | What was the duration of the inter views or focus group? | 42 minutes  (p10, line 215) |
| 22. Data saturation | Was data saturation discussed? | Given that the programme we evaluated was implemented over 5 years, each time we interviewed members the data added to our understanding of programme progress since the previous data collection |
| 23. Transcripts returned | Were transcripts returned to participants for comment and/or correction? | No |
| **Domain 3: analysis and ﬁndings** |  |  |
| *Data analysis* |  |  |
| 24. Number of data coders | How many data coders coded the data? | WO coded but then  discussed the code  list with the analysis  team.  (p12-3, lines 251-63) |
| 25. Description of the coding tree | Did authors provide a description of the coding tree? | Provided in  Supporting information S2 Fig |
| 26. Derivation of themes | Were themes identiﬁed in advance or derived from the data? | Derived  (p12-3, lines 260-3) |
| 27. Software | What software, if applicable, was used to manage the data? | Atlas.ti v8  (p12, line 254) |
| 28. Participant checking | Did participants provide feedback on the ﬁndings? | No checking was  done |
| *Reporting* |  |  |
| 29. Quotations presented | Were participant quotations presented to illustrate the themes/ﬁndings? Was each quotation identiﬁed? e.g. participant number | Yes, see Results  (p15-27) |
| 30. Data and ﬁndings consistent | Was there consistency between the data presented and the ﬁndings? | Yes |
| 31. Clarity of major themes | Were major themes clearly presented in the ﬁndings? | Yes, it is labelled as  Theme 1, 2 and 3  (lines 331, 371, 406) |
| 32. Clarity of minor themes | Is there a description of diverse cases or discussion of minor themes? | No minority themes  were identified; the  divergence between  well and less-well  performing teams is  the focus of the  Results. |
