# Supplementary figures and images for: “If we work as a team, there are success stories.” Unpacking team members’ perceptions and experiences of what impacts team performance in a maternal and neonatal quality improvement programme in South Africa, before, and during COVID-19
Source: PLOS Glob Public Health. 2024 Dec 23;4(12):e0003780. doi: 10.1371/journal.pgph.0003780 (PMC11665988; doi:10.1371/journal.pgph.0003780)

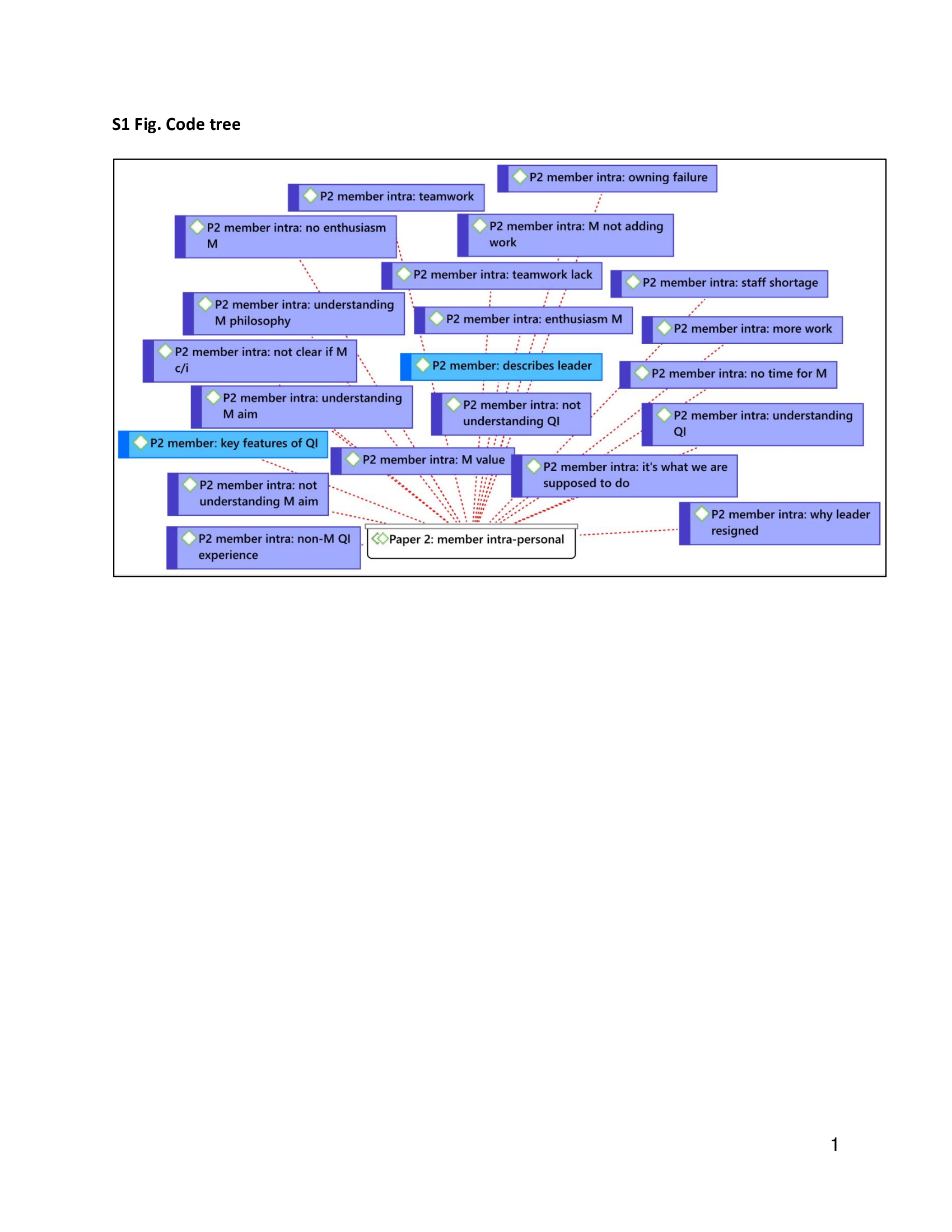

Supplement: S1 Fig — (TIFF) [file pgph.0003780.s003.tiff]
