# Supplementary material for: “If we work as a team, there are success stories.” Unpacking team members’ perceptions and experiences of what impacts team performance in a maternal and neonatal quality improvement programme in South Africa, before, and during COVID-19
Source: PLOS Glob Public Health. 2024 Dec 23;4(12):e0003780. doi: 10.1371/journal.pgph.0003780 (PMC11665988; doi:10.1371/journal.pgph.0003780)
Supplement: Attachment — Submitted filename: Response to Reviewers_24Jan24.pdf [file pgph.0003780.s004.pdf]

Response to reviewer comments
(24/01/24)

Submission ID
PGPH-D-23-01565

Title
"If we work as a team, there are success stories." Unpacking team members' perceptions and experiences of what impacts team performance in a maternal and neonatal quality improvement programme in South Africa, before, and during COVID-19

Dear Reviewers

Thank you for taking the time to review our manuscript. We are very grateful for your considerate and constructive suggestions that improved quality of the manuscript.

1. There are 4 instances where reviewing the manuscript resulted in us rephrasing text. These self-initiated changes are highlighted in yellow fill:
    - lines 66-7
    - line 75
    - line 319
    - line 441

2. The mentioned 'manuscript under review' has been published (reference 32), and we updated the text according, also highlighted in yellow fill:
    - lines 277-8
    - line 390

3. Kindly find our responses to your comments below.

Reviewer #1

Reviewer comment	Authors' response
1<br>This is an important topic and addresses the complex interlinked issues at play affecting the implementation and sustainability of quality improvement initiatives, particularly for maternity services, which can lead to barriers to success for these important initiatives.	Thank you.
2<br>a. In the description of the intervention it states that management selected 2-3 HCWs for training in each facility. Facility managers then selected the team leader from these trained HCWs. It would be good to understand more about who the participants were, how they were selected, and how and on what basis team leaders were chosen,<br><br>b. since composition of the teams is an important factor for WP teams. For example, was the selection based on seniority and experience? Was the selection process consistent across facilities?	2a<br>One of the co-authors, Shuaib Kauchali, who was involved in designing the Mphatlalatsane programme, responded to this comment, please see the details in the 'Personal communique' letter, attached to the resubmission. In short, the selection of trainees, leaders, members, and how to set up the teams, was purposively left to the facilities and teams, to advance local leadership and programme ownership. We amended the text to reflect this information:<br>• Setting section, lines 169-73.<br>• Discussion section, lines 616-21.<br>• Table 5, 'Training and setting up' recommendation<br><br>2b<br>We absolutely agree. Since we submitted this manuscript, our paper on the team leaders was published. We added our 'Leader paper' conclusion on the importance of leader characteristics, in the Discussion, lines 563-8.
3<br>a. Other team members mentioned include the second leader and the core team. How were these members selected and was this a designated part of the process of creating teams, or something that facilities themselves decided to do?	3a<br>• Lines 456-7: we did not find evidence of a standardised way in which it was done.<br>• Our response (2a) also applies here.

b. Similarly, the first criteria for WP teams was the presence of a leader and team structure – can you clarify what is meant by team structure and how this was defined.	3b<br>Lines 242-3: we clarified 'structure'.
4<br>As noted in the limitations, having leaders present in some of the interviews would very likely have affected participants responses. Even having another team member in the interview could have affected participant's responses if that person was the second leader or a member of the core team. A suggestion is to include in the brackets after each quote whether this quote is from an individual IDI, group interview or leader interview.	4<br>We do not think that adding this is going to tell us anything meaningful on how to interpret the quotes. This is because the influence of the leader's presence - if influential at all - could go in either direction. Either team members could have been more likely to highlight flaws and failings of QI in order to air their complaints in front of a leader, or felt restricted in their ability to highlight weaknesses. It is also possible that a mix of both sets of influence is applicable, and these would then 'cancel one another out' as sources of bias. Overall, given that we do not have data on what the relationship between team member participants in the group interviews were, it is not possible to say if and how this biased responses. We suggest keeping the leader - presence in interviews joint by team members as a limitation, but we also added not having information on the relationship between members in group interviews, as a limitation as it could have biased member responses, see lines 662-7.
5<br>Leaders role and skills – under this heading it could be made more clear (if I am correct) that all teams, both WP and LWP, were positive about their leaders roles and skills, suggesting that this is not an issue that differs between WP and LWP teams. However, all the quotes in this section are from WP teams.	5<br>The quote, line 337-8, is from an LWP team member. The difference between LWP and WP team members' views on leaders was that the latter elaborated on why they thought the leader was good, which was largely absent in LWP teams. Please see if the following changes clarify this sufficiently:<br>• line 339: add text to the quote from the WP team member<br>• lines 341-6: make it clear that characteristics of good leaders were largely absent in LWPs<br>• lines 663-7: postulate why LWP teams praised their leaders.

6<br>a. There is a major difference between the district 1-3 teams and the district 4 teams in terms of years of experience, which is highlighted in the text (L275). This suggests that the one facility in district 4 was very different from others. However, it is not clear, given that one of the teams in district 4 refused to participate, whether this one facility is the hospital or the clinic?<br><br>b. In addition, there were only five participants in district 4 (3 interviews). Please clarify whether this is indeed an important difference.	6a<br>It was the clinic, however, to protect the facility from being identified, those familiar with Mphatlalatsane know the facilities by name, we think it best not to say that the non-participating team came from the clinic.<br><br>6b<br>It is not important. There were on average 4 participants per facility, see lines 214-5 number across the 13 facilities. We also added the average participant number per district in Table 2.
7<br>The quote on L454 seems to suggest that LWP teams were also having a positive culture in their teams and there could be an additional comment to highlight this.	7<br>Thanks for the suggestion. We added text, lines 573-8, to clarify that LWP did at times display team characteristics we associated with the WP teams, but that these were inconsistently present.
8<br>L328 both the quote and the sentence before it are unclear	8<br>Lines 319-22: please see if the rewording clarifies it.
9<br>The longitudinal data collection is an important strength of this study as noted in the discussion, but this is not being utilized in the narrative. You could include some quotes that demonstrate how some of the themes and sub-themes changed over the data collection period. A series of quotes from the same facility over time for example.	9<br>We unfortunately do not have a good example mainly because we did not interview the same members over time, and were thus unable to track changes over time as reported by members.
10<br>In L460 it is noted that all well-performing teams had 3-6 core members taking charge.<br>a. It would be good to could give more explanation on how exactly how this was different in the LWP teams and<br><br>b. how the size of the facility was related to this, given that most of the smaller facilities (CHCs and clinics) were WP.	10a<br>Lines 457-9: please see if the addition clarifies it.<br><br>10b<br>In line 669 we mentioned that we did not collect data on actual team sizes as a limitation, and in lines 671-2 added that in the

	absence of actual team sizes, we did not infer if it was related to facility size.
11<br>In the conclusion the importance of team leadership is strongly highlighted as being key to success but the one thing that was common across WP and LWP teams was that they all praised their leaders.	11<br>Please see if the added text, lines 340-44 clarifies this. In lines 662-5 we offer possible reasons for this.
12<br>Small corrections<br>a. The first sentence of the introduction could be revised to read better – just a suggestion<br><br>b. The sentence about abandoning, adapting or adopting (L95) needs rephrasing<br><br>c. Rephrase an average of (L171)<br><br>d. Typo L223 remove and at the end of the sentence	12a<br>Thanks for the suggestion, please see if the change, lines 65-6 improves it.<br><br>12b<br>We amended line 92.<br><br>12c<br>Thanks, done, now line 167.<br><br>12d<br>We added ‘barriers’, now line 224.

Reviewer 2

Reviewer comment	Authors’ response
1<br>The study is about team members’ perspectives and experiences related to team performance in a quality improvement (QI) programme (Mphatlalatsane) implemented by the South African National Department of Health to reduce maternal/neonatal mortality and stillbirths, through improved quality care. The analysis provides a novel way of understanding the mixed results shown in other QI programmes by unpacking the differences between well and less-well performing teams. The usefulness of the study is that it identifies those factors	1-4<br>Thank you for the positive comments.

associated with successful QI team performance so that these can be replicated elsewhere.	
2<br>QI teams' performance was affected by existing facility relationships and contexts, how teams are set up, member mastery of the methodology, and the ability of a team leaders to obtain the trust, rapport with and support of the team, which could mean going beyond the training manual to inspire and model for their team, motivation for the program. Less well performing teams lacked sufficient understanding of program goals, had fractured intra-personal dynamics that exacerbated existing challenges such as staff shortages, attitudes towards work, Covid-19 and other trauma.	
3<br>While the teamwork aspects related to QI outcomes have been explored, the findings showcase specific institutional dynamics, for example the prevailing work culture, the team composition and the effects of Covid-19 on team performance.	
4<br>Table 5 offers immediately implementable recommendations for recruitment and set-up, training, and managing maternal and neonatal QIP teams in low-resource settings, which is a strength of the research. Given that the focus of the analysis was on understanding the drivers of difference between well- and less well-performing teams, it offers a road map to more successful QIP implementation.	
5<br>A thorough analysis of the themes is offered by the authors who used a 32-item checklist for reporting on qualitative studies. This makes the findings robust with the data and analysis offered supporting the claims made. Although it is noted that the lead author kept a fieldwork journal, it is not clear to what extent this influenced the data analysis.	5<br>Thanks for the observation. Please see if lines 651- address your comment.

6<br>a. The implications of improved quality of care on health outcomes was not fully grasped by less-well performing teams. Where teams chose to focus on partogram completeness but neglected to ascertain the way this tool should be used to facilitate decision-making, trigger preparations for transfer, discussions with senior nurses and the need for medical action - then the quality improvement associated with the task was poorly understood. This point could be given more attention in the article as monitoring and evaluation of maternal and foetal indicators must occur prospectively so that indications of the need for medial interventions can be deciphered timeously. Instead it seemed to be occurring retrospectively, stopping nurses from attending to patients when it was necessary, and hence the QIP may even be negatively associated with worsening health outcomes where the link between quality and improved mortality and morbidity was not well understood. This is a point that should be raised in the article.<br><br>b. Improvements in work processes are not always associated with improvements to patient care.	6a<br>We totally agree with your observation that in this case, the team did not make the link between regarding prospective indicators to trigger medical interventions, and added lines 546-8 to highlight it.<br><br>6b<br>We fully agree as stated in lines 601-6. Also, please note that assessing the clinical content of the QIPs, as per the partogram completeness was beyond the scope of evaluation, and this paper.
7<br>Line 575 "embedded in in existing" - repeated word	7<br>Thanks, removed, now line 583.
8<br>Line 599 "became a performance" - extra space	8<br>Corrected, line 606.
9<br>Line 223 "enablers and. The" - additional word	9<br>We added ‘barriers’, now line 224.
10<br>Line 400 "she did not having" - grammar	10<br>Corrected, now line 397.
11<br>Line 444 "service teams This". missing full stop	11<br>Added, now line 440.
